# Efficacy of Licorice Lollipops in Reducing Dental Caries in a Paediatric Population: A Systematic Review

**DOI:** 10.3290/j.ohpd.a44138

**Published:** 2020-04-01

**Authors:** Sivakumar Nuvvula, Mahesh Nunna, Merve E. Almaz, Sreekanth K. Mallineni

**Affiliations:** a Professor, Department of Paedodontics and Preventive Dentistry, Narayana Dental College, Nellore, Andhra Pradesh, India. Idea, hypothesis, risk of bias, review of the manuscript.; b Postgraduate Student, Department of Paedodontics and Preventive dentistry, Narayana Dental College, Nellore, Andhra Pradesh, India. Idea, data search, data analysis, risk of bias, preparation of manuscript.; c Assistant Professor, Department of Paediatric Dentistry, Faculty of Dentistry, Kırıkkale University, Turkey. Review of the literature, preparation and review of the manuscript.; d Associate Professor, Department of Paedodontics and Preventive dentistry, Narayana Dental College, Nellore, Andhra Pradesh, India; Associate Professor, Paediatric Dentistry, Department of Preventive Dental Science, College of Dentistry, Majmaah University, Majmaah, Saudi Arabia. data search, study design, data analysis, preparation and review of the manuscript.

**Keywords:** children, dental caries, licorice lollipops, *Streptococcus mutans*

## Abstract

**Purpose:**

To assess the efficacy of licorice lollipops in reducing dental caries in children.

**Materials and Methods:**

A literature search was confined to the English language using MeSH terms congruent with PICO format in ‘PubMed’, ‘Cochrane Library’ and ‘Ovid’, covering the period from April 1967 to December 2017. Searches in Google Scholar, grey literature and hand search of cross-references were performed to find additional data. Suitable studies were selected based on the predefined inclusion and exclusion criteria. Quality analysis and risk of bias of the selected studies were performed using the Cochrane Collaboration’s tool for risk of bias.

**Results:**

Overall 519 articles were retrieved, 516 (electronic databases) and 3 (Google scholar). 516 publications were excluded due to non-availability of abstracts, or because they were unrelated studies, narrative reviews, and systematic reviews as well as letters to editors. Only three studies were included for final analysis. Quality analysis of these three studies showed that only one was of high quality, whereas the other two were rated as low.

**Conclusion:**

Licorice lollipops showed a promising effect in reducing caries by decreasing *Streptococcus mutans* counts in the saliva. Further research using randomised controlled clinical trial (RCT) designs with large sample size are recommended.

Dental caries is the most commonly observed infectious oral disease in the world, and is highly prevalent in India as well.^23^ *Streptococcus mutans* (SM) is the principle cariogenic bacteria in the oral cavity.^5^ *Lactobacillus, Actinomyces* and *Velionella* species are other members of the microflora responsible for tooth decay.^24^ The production of acid, resulting from sugar metabolism by these bacteria and the subsequent decrease in environmental pH, is responsible for the demineralisation of the tooth surface.^14^ Episodes of repeated dissolutions lead to cavitation of the tooth surface, which should be treated at the earliest.^13^ Development of carious lesions shifts the microflora on the tooth surface from the dominance of non-mutans streptococci and *Actinomyces* to the dominance of SM.^21^ Considering the fact that tooth decay is an infectious disease, antimicrobial treatments against cariogenic bacteria should prevent it.^9^ Reducing the intake of cariogenic foods, application of topical fluorides, as well as pit and fissure sealants, are the routinely followed procedures for hindering caries progression.^7^ Recently, natural and herbal products such as cocoa, miswak, propolis, and tea leaves have been shown to have an anti-caries effect^11^ and demonstrated their antibacterial activity through the reduction of dental plaque formation.^18^

Licorice (‘mulethi’ in India) is a perennial herb in the legume family (Fabaceae) that grows up to 2 m height with long, cylindrical, thick, multi-branched roots.^14^ The oldest extant specimen was first introduced in the 8th century from China.^4^ Around 50% of Chinese herbal remedies contain various amounts of licorice.^4^ In India, the licorice root carries the ancient Sanskrit name of ‘Yasthimadhu’ (sweet-stalk) used widely in Ayurveda and other traditional medicines.^22^ Licorice can thwart dental caries by inhibiting the glycosyltransferase activity of SM.^18^ For centuries, its use in treating gastric illness, chronic hepatitis, rheumatoid arthritis, depression, and many other medical diseases has been widely documented.^3^ Licorice can be processed into various forms such as lozenges, mouthwashes and lollipops; of these, lollipops are the most effective.^25^ A sugar-free, orange-flavoured lollipop has been developed (C3 Jian, Intelliherb; Inglewood, CA, USA), containing an extract of licorice root that has been shown to target and kill SM in vitro.^6^ These lollipops, which come in different flavours and colors, are very appealing to children, who consume them for a recommended period of time.^16^ The main purpose of introducing these lollipops was to deliver a simple and effective way of fighting decay for young children who are at high caries risk.^17^

Previous studies^1,9,12,15,17,20^ reporting the anti-microbial effect of licorice lollipops involving both adult and paediatric populations showed acceptable results. Hence, the present study aimed to systematically evaluate the efficacy of licorice lollipops in decreasing caries in children. The study hypothesis was that licorice lollipops are effective in reducing caries in children.

## Materials and Methods

A detailed database search was performed in ‘PubMed’, ‘Cochrane Library’ and ‘Ovid’ covering the period from April 1967 to December 2017, and was limited to the English language. The MeSH terms included ‘child,’ ‘tooth,’ ‘glycyrrhiza,’ ‘candy,’ ‘lollipop,’ ‘dental caries,’ ‘efficacy’ and their synonyms with multiple combinations, using Boolean operators and truncations to expand and narrow the search based on the guidelines provided by the databases. Searches in Google Scholar and the grey literature as well as hand searches were performed on cross-references of the included and relevant studies to find additional studies. The populations, interventions, comparisons, and outcomes (PICO format) used for this systematic search are described in Table 1. Randomised clinical trials, prospective clinical trials performed involving healthy children in whom licorice lollipops were used to reduce caries, and studies published in the English language were included. Studies involving children with systemic illness and older individuals were excluded. Narrative reviews, systematic reviews, conference abstracts and letters to editors were also excluded before final eligibility was determined.

**Table 1:** MeSH terms conferring to PICO

PICO	Population	Intervention	Comparison	Outcome
Characteristics	Children, dentition	Licorice candies/ lollipops	Dental decay	Efficacy
MeSH terms	Child, tooth	Glycyrrhiza Candy Lollipop	Dental caries	Efficacy
Alternative terms	Pediatric Paediatric Pedodontic Paedodontic Preschool Adolescent Teen Minor Young Primary dentition Primary teeth Primary tooth Deciduous dentition Permanent dentition Permanent teeth Permanent tooth Milk teeth Milk tooth Baby teeth Baby tooth	Glycyrrhiza glabra Liquorice Liquorices Licorice Licorices Candies Confection Lollipop Lollipops	Carious dentin Dental white spot White spot	Quantitative method Qualitative method Evidence Reduction Effectiveness Determine comparison Comparative

After the search process, two researchers (M.N., M.A.) independently examined the titles of the initially retrieved papers. After exclusion of duplicates and irrelevant titles, abstracts were assessed. Abstracts which did not satisfy our selection criteria were excluded. The full text of the screened studies was included if at least one of the reviewers thought that the paper addressed the issue in question and methodology was in line with the inclusion and exclusion criteria. All the selected studies were read and evaluated for final eligibility independently by both of the researchers. The present study protocol was registered with the PROSPERO international prospective register of systematic reviews (PROSPERO 2018: CRD42018108667). Quality analysis and risk of bias in the included studies were performed using The Cochrane Collaboration’s tool for assessing risk of bias.^16^

## Results

**Fig 1:**
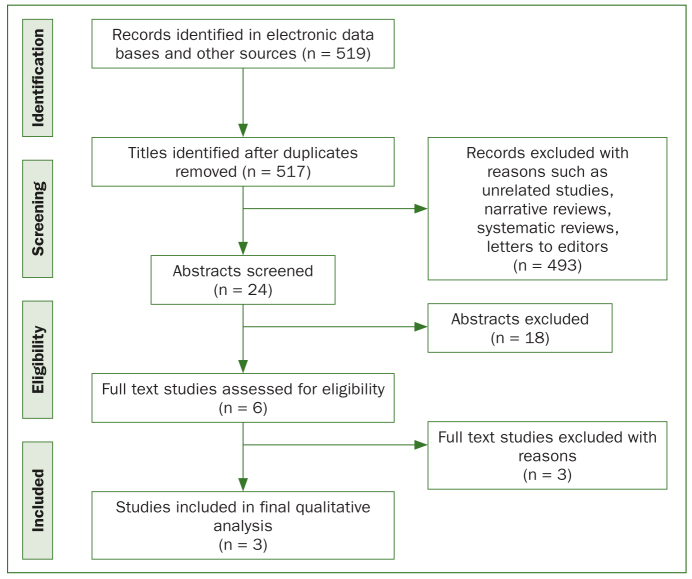
PRISMA flow diagram showing the comprehensive search process of the systematic review and identification of relevant studies.

**Fig 2:**
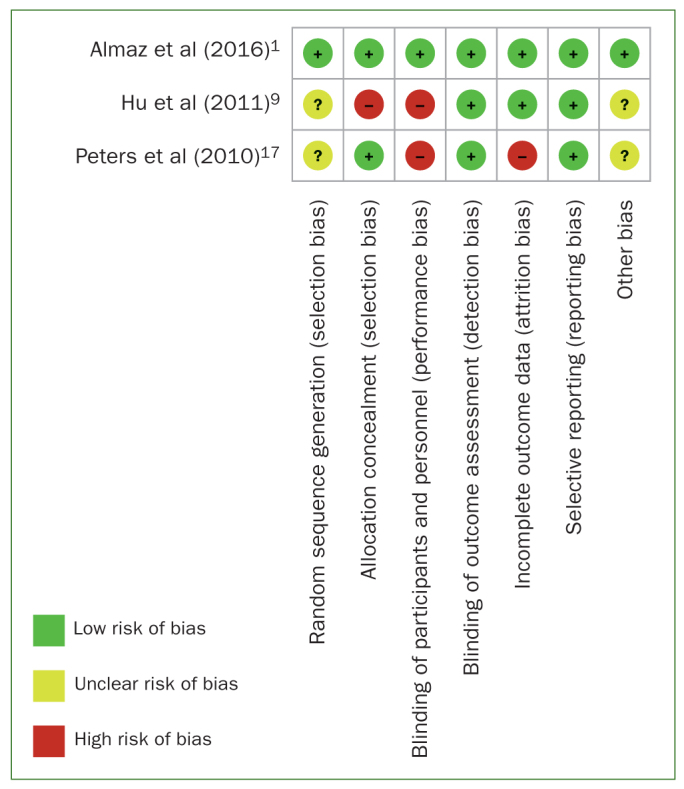
Risk of bias of included studies according to the Cochrane Collaboration’s tool for assessing risk of bias.

A total of 519 studies were retrieved from three databases (PubMed, Cochrane library, Ovid) and Google Scholar, of which 493 were excluded due to various reasons such as non-availability of abstracts, being irrelevant studies, narrative reviews or systematic reviews as well as letters to the editor. No relevant citations were found in the hand search. After screening 24 abstracts, 6 were assessed for full-text eligibility. Of these six, Mentes et al^15^ examined an older age group (85 years), Johnson et al^12^ focussed on children with asthma, and Srinathx et al^20^ with a sample size of n = 10 were excluded. Finally, 3 studies^1,9,17^ were thus included for quality analysis. A flow diagram of studies included for the review is presented (Fig 1) adhering to the Preferred Reporting Items for Systematic Review and Meta-Analysis Protocols (PRISMA-P).^16^ A kappa value of > 0.80 indicated substantial inter-examiner agreement for the methodological quality assessments for all categories.^24^ Details of the authors, study design, study groups, age, and sample size of the five initially evaluated studies are shown in Table 2. The intervention provided laboratory tests for *S. mutans* counts and statistical methods followed in the three included studies (Table 3). Almaz et al^1^ had a low risk of bias, while the remaining two studies^9,17^ exhibited high risk of bias according to the Cochrane Collaboration’s tool for assessing risk of bias (Fig 2).

**Table 2:** Details of initially included studies prior to comprehensive assessment

Author (year)	Study design	Study groups	Age (years)	Sample size	Included/ excluded
Almaz et al (2016)^1^	RCT	CF HCR (DT done) HCR (no DT)	5-11	108	Included
Hu et al (2011)^9^	2 CTs	LL	Different ages	26	Included
Johnson et al (2015)^12^	CCT	LL	4-16	50	Excluded
Mentes et al (2012)^15^	CT	LL	85	8	Excluded
Peters et al (2010)^17^	CT	HCR MCR LCR	2-5	66	Included
Srinath et al (2015)^20^	CCT	LL Controls	6-12	10	Excluded

RCT: randomised controlled clinical trial; CT: clinical trial; CCT: controlled clinical trial; CF: caries free; HCR; high caries risk; MCR: medium caries risk; LCR: low caries risk; DT: dental treatment; LL: licorice lollipops.

**Table 3:** Details of studies analysed

Studies	Year	Country	Intervention	Test	Statistical analysis
Peters et al^17^	2010	USA	Licorice lollipops twice daily for 3 weeks	GEE modelling	Wald test
Hu et al^9^	2011	USA	Licorice lollipops twice daily for 10 days	Monoclonal antibody test	Proc t-test
Almaz et al^1^	2016	Turkey	Licorice lollipops and placebo lollipops twice daily, 10 days for 3 months	Dentocult SM strip test	Chi-squared test

GEE: generalised estimating equations.

## Discussion

Caries progresses faster in children rate because of poor dietary habits and poor oral hygiene maintenance. Agents such as pit-and-fissure sealants and silver diamine fluoride to stop the progression of decay are now becoming increasingly popular. In this context, candies and lollipops from licorice extract show better results, as children prefer them. Studies performed on the effect of licorice in reducing caries suggested efficacy.^1,9,17^ A Turkish study performed by Almaz et al^1^ was the first randomised controlled clinical trial reported on the reduction of caries by licorice lollipops with the highest evidence; the remaining two studies^9,17^ were prospective clinical trials. All the three studies^1,9,17^ provided data on selection criteria of participants and settings. Peters et al^17^ conducted their clinical trial in an early ‘Head Start’ programme, whereas Almaz et al^1^ performed their study in children attending kindergarten, and the study by Hu et al^9^ was conducted in a university setting (UCLA, University of California, Los Angeles).

Licorice lollipops twice daily for ten days and three weeks were the intervention products used in studies performed by Hu et al^9^ and Peters et al,^17^ both in the USA. The study by Almaz et al^1^ used placebo lollipops in addition to the licorice lollipops twice daily for three months. The outcome measure in all 3 studies^1,9,17^ was a reduction of salivary *S. mutans* counts, with positive results in each trial. Peters et al^17^ reported no noteworthy changes in *S. mutans* levels in the low caries group after herbal lollipop use. Almaz et al^1^ stated that the *S. mutans* levels were not statistically significantly reduced after lollipop use in caries-free and high-caries-risk children. Hu et al^7^ attributed the anti-microbial activity of licorice to glycyrrhizol A, which kills strains of SM.

Only Almaz et al^1^ reported a priori justification for the sample size (the power was 0.80 with a significance level of 0.05). Power analysis was not performed in the studies by Peters et al^17^ and Hu et al,^9^ which accounts for the low rating in the quality analysis. In the study by Almaz et al,^1^ a double-blinded randomised controlled clinical trial from Turkey, children were asked to pick red or green papers and were accordingly allocated to placebo and herbal lollipop groups, respectively, by simple randomisation. Here, an experienced dentist carried out the implementation with her assistant.^1^ Peters et al^17^ distributed colour pamphlets to the Head Start children, and all were allowed to participate in the study. Hu et al^9^ followed no clear randomisation method.

All authors performed relevant statistical analyses in their studies (Table 3). In a high-caries-risk group of children using herbal lollipops, Almaz et al^1^ found a statistically significant reduction (p = 0.033) of *S. mutans*. In the study by Hu et al,^9^ a majority of participants exhibited substantial reductions of *S. mutans* after using licorice lollipops (p = 0.0002). The steepest early decrease in mean log-*Streptococcus mutans* (p < 0.001) in high-risk children was reported by Peters et al.^17^ Among the included studies,^1,9,17^ Almaz et al^1^ was the only study with a ‘high’ rating in the quality analysis, with a low risk of bias. All authors expressed their final results in terms of decreased CFUs of *S. mutans*.

Peters et al^17^ advised twice-daily use of licorice lollipops, which statistically significantly reduced the number and percent of *S. mutans* in high-risk children when used for 22 days. Hu et al^9^ reported that the consumption of licorice lollipops reduced a cariogenic bacterium, which helps to promote oral health in children and improve their quality of life. Licorice lollipops were found to be effective in high-caries-risk children (who did not comply with dental treatment) in reducing salivary *S. mutans* levels in the Almaz et al^1^ study.

Mentes et al^15^ reported a reduction of *S. mutans* in saliva after 21 days of lollipop use in 8 nursing home residents. In a pilot clinical trial by Srinath et al,^20^ licorice lollipops were found to be a good caries preventive strategy for children. Johnson et al^12^ advocated the reduction of caries in children who were taking beta-2 agonist drugs for asthma. Even though the results of these three studies^12,15,20^ were positive and satisfied our hypothesis, they were excluded for the following reasons: the Mentes et al study^15^ conducted in the US examined older individuals and had very small sample size (n = 8); an Indian pilot study^20^ had a sample size of only 10; the third study^12^ was done in children with a systemic illness. Since caries is a multifactorial disease, determination of only the *S. mutans* counts may not be sufficient to evaluate the anti-caries efficacy of licorice lollipops. Numerous studies have shown a strong relation between reducing cariogenic bacteria and reductions in dental decay. None of the above-mentioned clinical trials reported adverse events in children who consumed licorice lollipop.

Licorice has long been used in traditional medicine; subsequently, it was found that licorice is effective in caries prophylaxis. Recently, an India study^10^ reported that licorice extracts used in paediatric patients showed antimicrobial efficacy and led to a rise in the pH of saliva. The authors concluded that licorice extracts have antimicrobial and cariostatic efficacy and recommended that licorice can be used as a preventive regimen in paediatric practice.

One of the limitations of the present study was that it used only three databases, which could be the possible reason for weak evidence, along with the search being performed only in English language. Although the studies presented reliable, quality scientific evidence, some of them did not perform the sample size calculations, except Almaz et al.^1^ However, almost all the studies on licorice lollipops found them to promote caries reduction in children.

## Conclusions

1. With very minimal evidence obtained, licorice candies/lollipops showed a promising effect in reducing caries by decreasing the CFUs of *Streptococcus mutans* in saliva.
2. Licorice lollipops cannot completely replace preventive strategies such as proper dietary habits, maintaining good oral hygiene and fluoride application.
3. Further research with RCT designs comprising of large sample sizes should be carried out to discern the efficacy of licorice candies/lollipops in the prevention of dental caries.
